# The Construction of a Molecular Model for the Ternary Protein Complex of Intrinsic Coagulation Pathway Factors Provides Novel Insights for the Pathogenesis of Cross-Reactive Material Positive Coagulation Factor Mutations

**DOI:** 10.3390/ijms26115191

**Published:** 2025-05-28

**Authors:** Shifeng Jiang, Fang Li, Lei Li, Xuefeng Wang, Dongqing Wei, Wenman Wu, Qin Xu

**Affiliations:** 1 State Key Laboratory of Microbial Metabolism & Joint International Research Laboratory of Metabolic and Developmental Sciences, School of Life Sciences and Biotechnology, Shanghai Jiao Tong University, Shanghai 200240, China; jack-carpenter@sjtu.edu.cn (S.J.); lfsjtu@sjtu.edu.cn (F.L.); dqwei@sjtu.edu.cn (D.W.); 2 Department of Laboratory Medicine, Ruijin Hospital, Shanghai Jiao Tong University School of Medicine, Shanghai 200025, China; lilei.sjtu@alumni.sjtu.edu.cn (L.L.); xfwang63@shsmu.edu.cn (X.W.); 3 College of Health Science and Technology, Shanghai Jiao Tong University School of Medicine, Shanghai 200025, China

**Keywords:** FVIIIa-FIXa-FX (zymogen) ternary complex, intrinsic coagulation pathway, protein interactions, molecular modeling, molecular dynamics simulations

## Abstract

The human coagulation pathway orchestrates a complex series of events vital for maintaining vascular integrity, in which the intrinsic pathway plays a pivotal role in amplifying and propagating the coagulation response. Dysregulation of this pathway can lead to various bleeding disorders and thrombotic complications, posing significant health risks. In this pathway, the activation of Factor (F) X zymogen is catalyzed by the FVIIIa-FIXa binary complex, but knowledge about this is still incomplete. Understanding the structural and functional intricacies of the FVIIIa-FIXa-FX (zymogen) complex is imperative for unraveling the molecular mechanisms underlying coagulation regulation and guiding the development of targeted therapeutic interventions. In this study, utilizing Alphafold-Multimer and molecular dynamics (MD) simulations, we provide insights into factor interactions within the ternary complex and propose novel functional mechanisms contributing to the functional defects inflicted by their cross-reactive material (CRM) positive mutations. The amino acid residue replacement impairs the coagulation function by interfering with structure elements, including the following: (1) a knot-like structure between Arg-562 of FVIIIa’s 558-Loop (residue 555–571) and the 333-Loop of FIXa (residue 333–346) contributes to FVIIIa-FIXa binding; (2) the a2 region of FVIIIa (residue 716–740) opens the lid of active site (FIXa’s 266-Loop, residue 256–270) and facilitates substrate binding; (3) the activation peptide (AP) of FX zymogen (residue 143–194) not only assists in the activation of itself but also adheres the interface of the three factors like a double-sided tape. Our work provides novel insights for the pathogenesis of a number of reported clinical CRM-positive mutations and may lay the groundwork for the structure-based development of therapeutic interventions.

## 1. Introduction

The human coagulation pathway, fundamental for hemostasis and thrombosis regulation, encompasses a multifaceted cascade of events intricately orchestrated to maintain vascular integrity. The whole coagulation pathway consists of three parts: intrinsic, extrinsic, and common pathways [1,2,3,4]. The intrinsic pathway serves as a vital arm of the coagulation cascade, initiated within the bloodstream itself. The intrinsic pathway’s significance lies in its ability to amplify and propagate the coagulation response through the sequential activation of various coagulation factors, resulting in the formation of a stable fibrin clot. Unlike the extrinsic pathway, which is triggered by tissue factor exposed upon vessel injury, the intrinsic pathway is activated by contact with exposed subendothelial surfaces or negatively charged molecules, thereby serving as a critical part of the body’s defense mechanism against bleeding [5,6].

Among the key players in the intrinsic pathway are coagulation factors VIII, IX, XI, and XII. They are regulated to ensure hemostasis while preventing thrombotic complications [1]. Dysregulation of the intrinsic pathway can precipitate a myriad of disorders, including bleeding diatheses and thrombotic events. Hemophilia A or B, characterized by functional deficiencies in factors VIII (FVIII) or IX (FIX), are typical examples of intrinsic pathway dysfunction, manifesting as recurrent, often spontaneous, bleeding episodes [7,8,9,10]. Conversely, hypercoagulable states associated with excessive intrinsic pathway activation contribute to thrombotic disorders such as deep vein thrombosis and pulmonary embolism, posing substantial morbidity and mortality risks [11,12,13].

The FVIIIa-FIXa-FX (zymogen) ternary complex is composed of activated FVIII (FVIIIa), FIX (FIXa) (combined as intrinsic FX zymogen-activating complex), and FX zymogen. This complex is directly involved in the conversion of FX zymogen to the active form, FXa, thereby amplifying the coagulation response. FVIII functions as a cofactor of FIXa, and it significantly enhances the proteolytic activity of FIXa by five orders of magnitude [14,15]. Understanding the roles of these three components within the complex is fundamental to unraveling the intricacies of clot formation. Previously, several wet lab investigations were carried out that were dedicated to deciphering these interactions and have provided possible contacting residues and interfaces among the factors [16,17,18,19,20,21]. These findings trigger more and deeper research on the molecular basis of coagulation. The structure of the ternary complex serves as a focal point for research efforts aimed at elucidating factor–factor interactions. Several studies on the structure determination of single factors have been carried out so far. To date, 10 structures of FVIII, more than 40 structures of FIX, and more than 100 structures of FX have been resolved and uploaded to the Protein Data Bank [22,23,24]. Among these structures, the relatively intact structures with higher resolution are PDB entry 2R7E for FVIII [25], 2WPH for FIX [26], and 2Y5F for FX [27]. Despite these efforts, little is known about the structure of the whole FVIIIa-FIXa-FX (zymogen) complex. Given the pivotal role of the intrinsic pathway in hemostasis and thrombosis, elucidating the molecular basis of the FVIIIa-FIXa-FX (zymogen) complex is of great clinical importance. Structural insights into the whole FVIIIa-FIXa-FX (zymogen) complex are indispensable to unravel the pathophysiology of coagulation disorders, particularly those caused by cross-reactive material (CRM) positive mutations, which impair or enhance the function of coagulation factors but have little impact on their expression and secretion. The structural studies also provide a guide to the development of targeted therapeutic interventions, including several rational drug design cases targeting coagulation factors [28,29,30,31,32,33,34,35].

Due to the vast application of AI techniques to computational structural biology, the past ten years witnessed the boom of in silico tools in terms of macromolecular structure prediction and assembly. In this study, we delve into the structural aspects of the FVIIIa-FIXa-FX (zymogen) complex, aiming to contribute to a detailed understanding of interactions between FVIII, FIX, and FX. We provide the molecular basis for some frequently discussed CRM (+) mutations of the coagulation factors and propose functions and mechanisms within the complex. Through our study, we aspire to trigger more innovative structure-based drug/therapeutic design in managing thrombotic and hemorrhagic conditions.

## 2. Results

### 2.1. The Overall Structure of the Ternary Complex Model

During our early attempts to build the whole FVIIIa-FIXa-FX (zymogen) complex with AlphaFold-Multimer, we found that the results did not align with the wet lab findings. We hypothesized that AlphaFold-Multimer mistook FX zymogen for the sequentially similar FIXa in the MSA (Multiple Sequence Alignment) process. By checking the template for building the ternary complex, we found that the construction is based on a cryo-EM structure of human prothrombin-prothrombinase (PDB entry: 7TPP), involving FVa, FXa, and FII zymogen as the substrate in the common coagulation pathway. In this ternary complex structure, FXa catalyzes the activation of FII. The template information explains the reason for the above-mentioned mistakes during the construction of our ternary complex, as FX is recognized as the activated enzyme rather than the substrate. Importantly, as FII zymogen shares little sequential and structural similarity with FX zymogen, it is inappropriate to use AlphaFold-Multimer for ternary complex construction in a single step, and manual adjustment of FX zymogen conformation in our complex is necessary. These structural differences imply discrepancies in molecular mechanisms between intrinsic and common pathways, despite their similarities. Therefore, we turned to using AlphaFold-Multimer for intrinsic tenase complex (FVIIIa-FIXa binary complex) construction before introducing the FX zymogen to the system. One round of modification for binary complex modeling and seven rounds of main modifications for ternary complex construction have been conducted based on the literature and structural comparison (Table S1).

**Figure 1:**
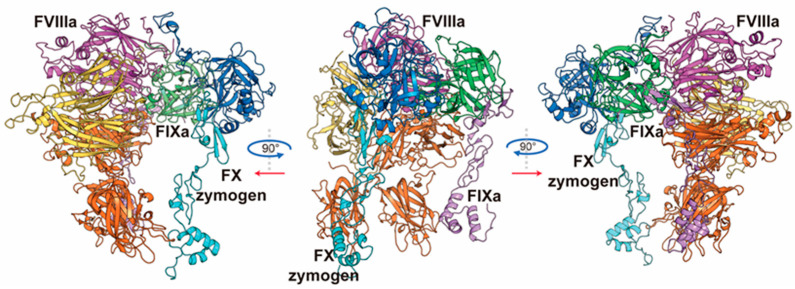
The overall structure of the FVIIIa-FIXa-FX (zymogen) ternary complex. For FVIIIa, domains A1 and A2 are colored in yellow and purple, respectively. Domains A3, C1, and C2 are collectively colored in orange. For FIXa, the light chain and heavy chain are colored in violet and green, respectively. For FX zymogen, the light chain and heavy chain are colored in cyan and blue, respectively.

The final model (Model 7-stable, Structure S1.pdb in the Supplementary Materials) is a ternary complex involving FVIIIa, FIXa, and FX zymogen. As shown in Figure 1, FVIIIa contains domains A1 (residue 1-336, yellow), A2 (residue 373-716, purple), A3 (residue 1692-2021, orange), C1 (2022-2171, orange), and C2 (2172-2332, orange). Sequentially following the A1 and A2 domains are FVIIIa’s a1 (residue 337-372, yellow) and a2 (residue 717-740, purple) regions, which are relatively flexible regions serving as linkers in FVIII zymogen. FIXa involves both an intact light chain (residue 1-137, violet) and heavy chain (residue 181-415, green). FX zymogen is constructed with a light chain (residue 1-139, cyan) and heavy chain (residue 143-448, blue), along with an activation peptide (residue 143-194, hereinafter referred to as AP). MD simulation trajectories show stable conformation of each component, especially for the ternary interface involving FVIIIa’s A2 domain and the heavy chains of FIXa and FX zymogen. The main dynamic conformational changes lie in the light chains of FIXa and FX zymogen, as well as in FVIIIa’s C1 and C2 domain, especially in the terminal regions (Figure 1, Figures S1 and S2). With the AP of FX zymogen in the substrate pocket of FIXa, this complex demonstrates the pre-activation state of FX zymogen. The complex exhibits structurally reasonable backbone dihedral angles, as indicated by the Ramachandran plot (Figure S3).

In Model 7-stable, FIXa mainly binds to the side of FVIIIa in a stable manner. Interestingly, although we used Alphafold-Multimer, a different method from that of a previous work for the construction of the FVIIIa-FIXa binary complex [36], the contact surfaces between FVIIIa and FIXa in both structures are similar: the EGF domains as well as the GLA domain of FIXa form stable contact with FVIIIa’s A3 and C1 domains, and the SP domain of FIXa mainly possesses the binding surface with A2 of FVIIIa. Furthermore, in another study of ours currently under review, involving the construction of an FVIIIa-FIXa binary complex using protein–protein docking, the interactive parts align well with these findings. These results suggest that our proposed binding conformation of FVIIIa and FIXa provides rather reliable insight into the ternary complex. Although other contact surfaces of FVIIIa were also proposed in previous publications based on some experimental results, such as those in the C2 domain [37,38] and region 484–509 [39], more reasonable key contact regions of FVIIIa from other experimental investigations are included in our proposed model, such as regions 556–565 [18,19,20,40], 709–713 [41], 1790–1798 [42], and 1811–1818 [43]. These regions were generally characterized in experiments comparing relevant enzymatic kinetic data for FXa generation in blood systems involving FIXa, FX zymogen, and different FVIIIa fragments. But in some studies, the interaction regions were determined through analytical methods such as surface plasmon resonance (SPR) and enzyme-linked immunosorbent assay (ELISA). We carefully checked the experimentally identified contact regions of FVIIIa together with the analytical methods and confidently hypothesize that those partial regions not involved in our model might interact with FIXa or FX zymogen but might not be crucial to the coagulation activation of the FVIIIa-FIXa-FX (zymogen) complex. For instance, in previous research involving SAXS analysis of the intrinsic tenase complex [16], the conformation of the FIXa-FVIIIa complex in the model is unfavorable for the binding of FX zymogen in that FIXa is obviously distant from FVIIIa, and the binary complex does not provide enough space for FX zymogen docking and potential substrate insertion (Figure S4).

Both the heavy and light chains of FX zymogen are also stabilized in the ternary complex. For the light chain, the GLA domain of FX zymogen is stabilized by the FVIIIa C2 domain, which is the site for membrane anchoring for both coagulation factors. The SP domain of FX zymogen forms a stable contact surface with both FVIIIa and FIXa (details presented in the following sections).

### 2.2. The Critical Interaction Between Arg-562 of FVIIIa’s 558-Loop and the 333-Loop of FIXa Contributes to the Protection of FVIIIa from FXa Cleavage

**Figure 2:**
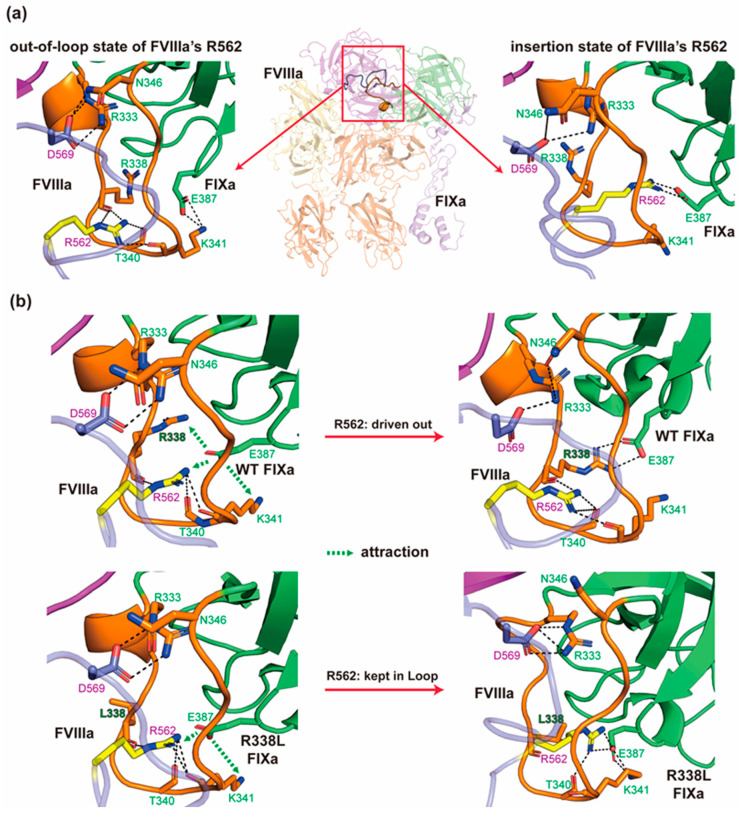
The binding interface between FVIIIa’s 558-Loop and the 333-Loop of FIXa. (**a**) The interaction between the 333-Loop of FIXa and the 558-Helix of FVIIIa. (**b**) The dynamic conformational changes in the 333-Loop of FIXa wild type and R338L (Pauda variant). The orange loop indicates FIXa’s 333-Loop, while the cartoon in green represents the rest of FIXa. The transparent blue loop represents FVIIIa’s 558-Loop, while the opaque cartoon in purple represents other parts of FVIIIa. The important residues are colored same as their backbone cartoon except that R562 of FVIIIa is colored in yellow.

It has been reported in previous experimental studies and also widely recognized that the interaction of the 558-Loop of FVIIIa and 338-Helix of FIXa plays an important role in the binding of FIXa [18]. However, the molecular mechanism behind it remains to be revealed. In our proposed model, the 338-Helix (residue 333–338) of FIXa together with its sequentially neighboring residues 339–346 form a giant loop (hereinafter referred to as a 333-Loop) that binds to the 558-Loop (residue 555–571) of FVIII via several hydrogen bonds with the main chain oxygen atoms of the loop (Figure 2a, left panel). For the upper part of the 333-Loop that is closer to the K293-Helix (c126-Helix in chymotrypsin numbering), R333 and N346 of FIXa, along with D569 of FVIIIa form a local interaction network: the side chain of FVIIIa D569 attracts the guanidine group and the amino group of R333 and N346. Meanwhile, D569’s nitrogen atom from its main chain forms a hydrogen bond with the acyl group of N346. These interactions form a knot-like structure to stabilize the upper part of the 333-Loop of FIXa. For the lower part of the 333-Loop, the main chain acyl groups surround and attract the R562 guanidine group of FVIIIa, thus strengthening the binding of FIXa to FVIIIa. Interestingly, during the MD simulations of Model 6 (Model 6-out of loop), one trajectory shows that the side chain of FVIIIa’s R562 passes through the loop to the other side and forms a salt bridge with E387 of FIXa (Figure 2a, right panel). This replaces E387’s former intra-molecular salt bridges with R338 and K341. E387 serves as an anchor in a manner similar to D359 (cD189) for substrate binding in the catalytic pocket of FIXa (shown in Section 2.4.) that further strengthens the binding of FIXa to FVIIIa. This is an amazing phenomenon, as it might provide a new molecular pattern of protein–protein binding: knotting. Although knotted proteins are attracting academic interests more and more, earlier studies mainly focused on the structure, folding mechanism, or even design of a knotted protein in a single chain [44,45,46]. This is the first time a reversible knot-like structure formed between two proteins through a non-covalent bond is reported and emphasized for protein–protein binding, as far as we know.

Furthermore, FIXa’s R338 is an intriguing site where several variants were clinically and experimentally identified with improved clotting activity [47,48]. For example, the famous R338L Padua hyperactive FIXa variant still has its molecular mechanism unrevealed [49]. Therefore, we further simulated and compared the conformational changes of R562 regarding 333-Loop in the ternary complexes of wild-type FIXa or R338L mutant, using Model 7—containing an in-loop state of R562—as the starting structure (herein referred to as Model 7-in loop or Model 7-R338L-in loop, Figure 2b). In two out of three replicated trajectories of the R338L variant, the absence of the positively charged side chain of L338 provides better chances for the E387 of mutant FIXa to attract the R562 of FVIIIa (Figure 2b, lower panel). This interaction is not as stable as that of FX zymogen’s R194 and FIXa’s D359 (cD189) because (1) there is still space for the dynamic flexibility of FVIIIa’s R562, which is created between region S384-C389 (c215-c220), and the 333-Loop of FIXa, forming a stable concave for FVIIIa’s R562 as a result of structural rigidity supported by a C361-C389 (cC191-cC220) disulfide bond, C336-C350 (cC168-cC182), and the neighboring rigid residues; (2) the R338 and K341 of FIXa compete with the R562 of FVIIIa for this electrostatic attraction (Figure 2b and Figure S5). This unstable salt bridge might be critical for the dissociation of the FVIIIa-FIXa binary complex in physiological conditions. In contrast, in two out of the three replicated trajectories of the complexes with wild-type FIXa, the R338 or K341 of FIXa competes with the R562 of FVIIIa for the electrostatic attraction by E387, which leaves R562 unstable and drives it back out of the 333-Loop (Figure 2b, upper panel). The other trajectory shows the maintenance of the ‘in loop’ conformation of FVIIIa’s R562 despite FIXa’s K341 maintaining the salt bridge with E387 at the same time. This suggests that FIXa’s R338 is the main competitor with R562 for the salt bridge, and higher efficacy may result from double mutants of R338 and K341. From the comparison between the MD simulations of R338L mutant and wild-type FIXa, we propose that the local electrostatic interactions assure the insertion and exclusion of FVIIIa’s R562 through FIXa’s 333-Loop, and the R338L variant tends to maintain the inserted state of R562. This hypothetical mechanism may explain the multiple reported mutants of R338 (R338L, R338P, R338Q), K341 (K341E, K341N), and E387 (E387Q, E387K, E387A, E387G) in the Factor IX Gene (F9) Variant Database (http://www.factorix.org/index.php (accessed on 11 Mar 2024)) [50].

Taken together, our proposed model provides a new insight for the interaction between FIXa and FVIIIa, especially for the interaction between the 333-Loop and 558-Loop. We believe that the superactive R338L mutation may boost intrinsic coagulation activation not only by the stabilization of the interaction between FVIIIa and FIXa, but also by protection on FVIIIa. In the human body, it was reported that FVIIIa’s is cleaved by FXa at R562 under physiological conditions [51], which would reduce its half-life in blood circulation and limit its overall efficiency as the cofactor of FIXa. Based on our models and MD simulations, we hypothesize that FIXa’s 333-Loop can accommodate and stabilize FVIIIa’s R562 and 558-Loop, therefore protecting part of the FVIIIa molecules from degradation, maintain the quantity of FVIIIa in the bloodstream, and prolonging its half-life. Compared with the wild type, our MD simulations on the FIXa R338L variant show improved stability of the knot-like structure at FIXa’s 333-Loop and reduced exposure of FVIIIa’s 558-Loop. Therefore, both the stability of the intrinsic tenase and the protection on FVIIIa from degradation could benefit from this R338L mutation. This hypothesis may provide a molecular mechanism to explain the hypermutation R338L and contribute to future drug development for hemophilia through protein engineering.

### 2.3. The a2 Region of FVIIIa Drives the Opening of FIXa’s 266-Loop (c99-Loop) and Maintains Its Open State

**Figure 3:**
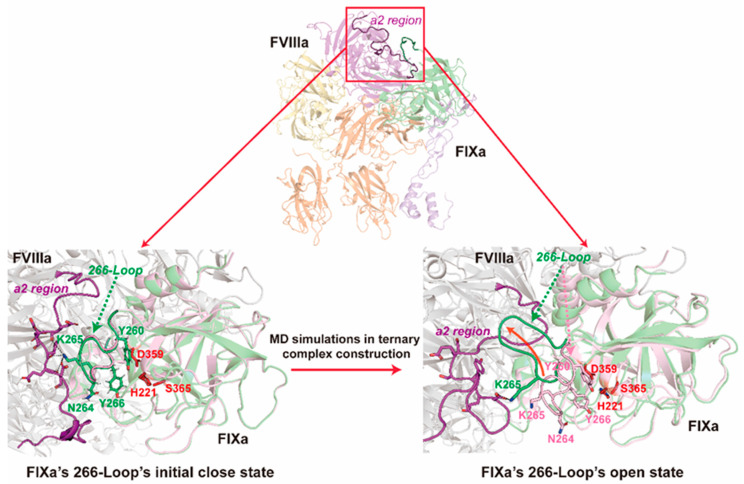
The “pulling out” of FIXa’s 266-Loop (c99-Loop) in the FVIIIa-FIXa-FX (zymogen) ternary complex. For clear representation, only FVIIIa and FIXa are shown in the figure. The pink ribbon indicates the initial conformation of FIXa in MD simulation, while the green ribbon represents the conformation of FIXa after MD simulation. The purple loop represents the a2 region of FVIIIa. The red residues denote the catalytic triad of FIXa.

During the construction process of our ternary complex model, another intriguing phenomenon was discovered regarding the 266-Loop (residue 256–270, also known as c99-Loop) of FIXa. The interaction of FIXa’s 266-Loop with FVIIIa’s a2 region (residue 716–740), a relatively disordered region at the C-terminal of the A2 domain, was modeled by AlphaFold-Multimer when we were constructing the binary complex structure (Figure S6). In this binary complex model (Model FIXa-FVIIIa in Table S1), despite the improperly modeled C-terminal residues that were placed into the catalytic pocket of FIXa, K265 (cK98) of the FIXa’s 266-Loop is electrostatically attracted by segment E720-D725 (amino acid sequence: EDSYED) of FVIIIa. These negatively charged residues form an arm-like conformation that surrounds the positive K265. Based on this structure, we retained the contacting part of the a2 region of FVIIIa and modified the C-terminal by placing it out of FIXa’s catalytic pocket and optimizing the whole binary complex with MD simulations (Model MOD1-FVIIIa-FIXa in Table S1). The trajectories show that the 266-Loop is a little pulled away from the SP domain by electrostatic attraction from the E720-D725 of FVIIIa on the residue K265 (cK98). Later, we established the subsequent ternary FVIIIa-FIXa-FX (zymogen) complexes with no modification at this contact region. In the simulations of multiple ternary models (Model 1 and Model 2), FIXa’s 266-Loop is evidently ‘pulled out’ by FVIIIa’s a2 region and maintained in an open state in the rest of models (Figure 3 and Figure S7). The open state of FIXa’s 266-Loop removes the steric hindrance posed by the side chains of Y260 (cY94), N264 (cN97), and Y266 (cY99) near the catalytic triad that blocks the entering of the long FX zymogen’s AP, thus facilitating the catalysis by FIXa. In contrast to this conformational change, all three trajectories of MD simulations of wild-type FIXa monomer (constructed from PDB entry: 3LC3) show the maintenance of a closed state with little dynamics in the 266-Loop (Figure S7). These results lead to our proposition that the binding of the cofactor FVIIIa to FIXa may cause the conformational change in its 266-Loop and facilitate the binding and the following cleavage of FX zymogen’s AP in FIXa’s active site. Therefore, our model may demonstrate the molecular mechanism responsible for how FVIIIa boosts the activity of FIXa for FX zymogen activation, at least partially.

### 2.4. FX Zymogen Contributes to Its Binding to FIXa and Facilitates the Activation of Itself in the Ternary Complex

**Figure 4:**
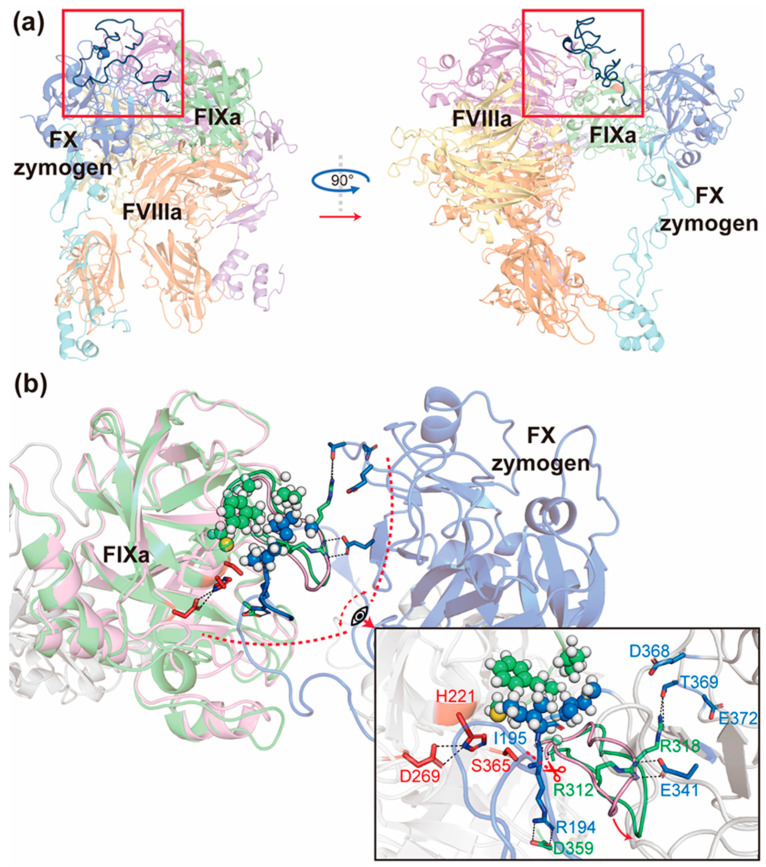
FX zymogen’s AP binding to the catalytic pocket of FIXa. (**a**) The position of FX zymogen’s AP within the entire ternary complex. (**b**) The local conformational changes in FX zymogen and FIXa during MD simulation. The pink ribbon indicates the initial conformation of FIXa in MD simulation, while the green ribbon represents the conformation of FIXa after MD simulation. The blue ribbon represents a representative conformation of FX zymogen. The red residues are the catalytic triad of FIXa, while spheres represent the hydrophobic contacting residues. The red arrow indicates the important conformational change of FIXa around the active site upon the binding of FX zymogen’s AP. And the red scissors indicates the peptidyl bond of AP to be cleaved in FX activation.

After pre-mature cleavage at the N-terminal, the FX zymogen’s AP in its predominant plasma-circulating form is a 52-amino-acid flexible peptide connecting the hydrophobic N-terminal of FXa (amino acid sequence: IVGG) [52]. So far, there has been no complete FX zymogen AP-bounded FIXa structure added to the Protein Data Bank. Therefore, we referred to a FIXa structure that binds to an amino acid mimetic substrate (PDB entry: 2WPI) in order to model the interaction of FX zymogen’s AP and FIXa catalytic site. In our proposed model (Figure 4), FX zymogen’s AP is inserted into the catalytic pocket of FIXa, with the catalytic residues H221 (cH57) and S365 (cS195) close to the amide C-N bond of R194 and I195 of AP. The guanidine group of FX zymogen’s R194 is stabilized by a salt bridge with D359 (cD189) in the cleft at the bottom of FIXa’s catalytic pocket, which provides an anchor for the AP to dock into the pocket. This conformation shows a pre-cleavage state of FX zymogen activation.

Using this structure as the starting conformation, further MD simulations (Model 7) show conformational changes that reveal the binding mechanism of FX zymogen’s AP. First, the FIXa’s autolysis loop (residue 310–323, c141–c155) is slightly pulled away from the catalytic triad due to electrostatic attraction or the hydrogen bond provided by the SP domain of FX zymogen. Specifically, R312 (cR143) and R318 (cR150) in the loop are attracted by FX zymogen’s E341 or E372, and T369 or D368, respectively. These interactions remove the steric hindrance limiting the substrate to access the catalytic pocket. Second, the side chains of the three remaining N-terminal residues after substrate cleavage (amino acid sequence: IVG) bind to the adjacent inner surface of FIXa’s 199-Loop (residue 199–205, c34–c41) as well as FIXa’s G363 (cG193) via stable hydrophobic interaction (spheres in Figure 4b). These two conformational changes, along with the anchor role of D359 (cD189), demonstrate possible aspects of substrate binding that might take place simultaneously or sequentially: with the stabilization and hindrance removal by the SP domain of FX zymogen, the above-mentioned hydrophobic residues of FIXa and FX zymogen assist the AP to find a proper pose to form the salt bridge with the anchor residue D359 (cD189) of FIXa. Through these interactions together, the FX zymogen’s AP is stabilized conformationally and prepared for cleavage.

### 2.5. FX Zymogen’s AP Stabilizes the Ternary Complex by Providing Double-Sided Interactions at the Interface of the Three Factors

**Figure 5:**
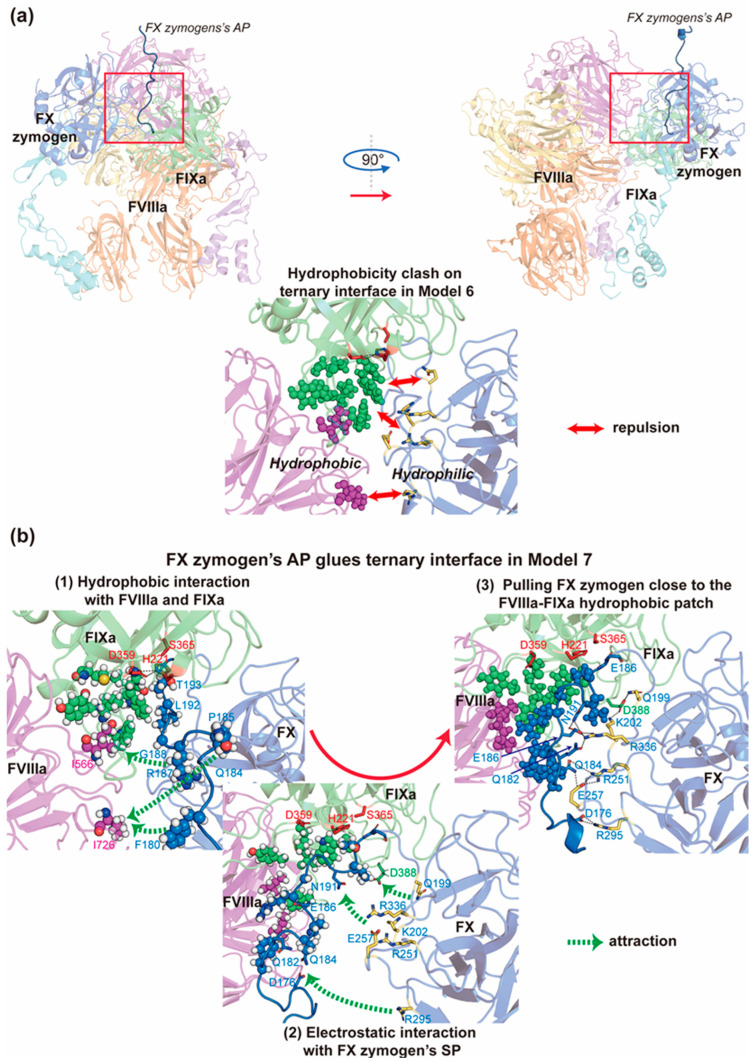
The function of the double-sided interactions of FX zymogen’s AP. (**a**) The hydrophobicity clash on the interface of FVIIIa-FIXa and FX zymogen. (**b**) The dynamic changes in FX zymogen’s AP gluing the interface during the MD simulation of Model 7. FVIIIa, FIXa, and FX zymogen are colored in purple, green and blue, respectively. The red residues denote the catalytic triad of FIXa, yellow residues represent charged residues, and the spheres represent hydrophobic residues. Red double-headed arrows indicate repulsion, while green dashed arrows indicate attraction.

One critical issue regarding the efficient cleavage of FX zymogen’s AP in a physiological context is the molecular basis for stabilization of all components in the complex. However, an intriguing phenomenon regarding the interactive surface of the three coagulation factors is that the FVIIIa A2 domain and part of FIXa’s SP domain are structurally proximate and form a hydrophobic patch, while FX zymogen to be catalyzed contains several charged and hydrophilic residues on the contacting surface to bind it (Figure 5a). This inconsistency of hydrophobicity was not solved in Model 4 and Model 6 (Model 6-out of loop, see Figure S8) with several rounds of modifications (Table S2) on the a1 region of FVIIIa (residue 337–372). This region was not determined in known crystal structures of FVIIIa and may be improperly predicted by AlphaFold2-Multimer in the beginning (over 75% residues with pLDDT < 30, Figure S6), where it contacts FX zymogen’s EGF2 domain in a manner different from the corresponding regions of our template (PDB entry: 7TPP). In addition, a previous experimental study [53] on FVIIIa’s a1 region indicates that it interacts with FX zymogen’s heavy chain instead of the EGF2 domain in the absence of phospholipid and FIXa. Taken together, we hypothesized that the a1 region might somewhat contribute to the preliminary approximation of FX zymogen to FVIIIa but may be not helpful for the final stabilization of the ternary complex in the pre-activation stage. Therefore, we removed FVIIIa’s a1 region and constructed the starting conformation of Model 7 (Model 7-out of loop) with a flexible N-terminal AP of FX zymogen (residue 143–190) extended in solution (same as the AP conformation in Model 6-out of loop) so as to search for possible conformations through extensive MD simulations.

In “Model 7-out of loop”, the three parallel 300 ns MD simulation trajectories demonstrate extensive conformational changes in the FX zymogen. In trajectory 1, the flexible N-terminal AP swings around the cleft between the FVIIIa’s A2 domain and FX zymogen’s SP domain in the first 50 ns before it forms relatively stable hydrophobic interaction with the above-mentioned hydrophobic patch of the FVIIIa and FIXa interface. In the next 100 ns, the N-terminal AP pushes the SP domain of FX zymogen a little away from the FVIIIa’s A2 domain as a result of steric hindrance and then forms stable salt bridges through several charged amino acid residues on the other side of AP in the last 150 ns. In trajectory 2, the N-terminal AP swings back and forth intensively in the solution, and the SP domain repeats an intriguing pattern of approaching and leaving the FVIIIa-FIXa interface due to the inconsistency of hydrophobicity in the contacting surface of FX zymogen. In trajectory 3, the AP also forms a two-sided interaction between FVIIIa and FX zymogen, but in a relatively flexible way due to the interaction between FVIIIa A2’s N-terminal peptide and the AP of FX zymogen. Taken together, the results above all may suggest that the highly flexible AP experiences a dramatic conformational change so as to fulfill the main gap between the hydrophobic patch of FVIIIa-FIXa and the hydrophilic contact surface of FX zymogen, which plays an important role in the stabilization of the ternary interface.

In addition, we extended trajectory 1 for another 200 ns of MD simulation in three replicates to check the stability of this ternary complex. Consistently, all components in the trajectories are relatively stable in the last 100 ns (Figures S1 and S2). This final structure is referred to as Model 7-stable (Figure 1), where the N-terminal AP of FX zymogen stably glues the gap on the interface of the three coagulation factors through double-sided interactions (Figure 5b and Table S2). On one side, the side chains of hydrophobic residues F180, P185, and G188 as well as the hydrophobic groups or parts of R187, Q184, and T193 form a long hydrophobic area that interacts with the FVIIIa-FIXa interface involving residues of the 387-Loop (residue 387–394, c217-c224) and the 333-Loop of FIXa. On the other side, via D176, Q182, Q184, E186, and N191, the AP forms an electrostatic network that stabilizes the SP domain of FX zymogen as well as part of the 387-Loop of FIXa.

### 2.6. A Detailed Proposal of the Assembly Process of FVIIIa-FIXa-FX (Zymogen) Complex

**Figure 6:**
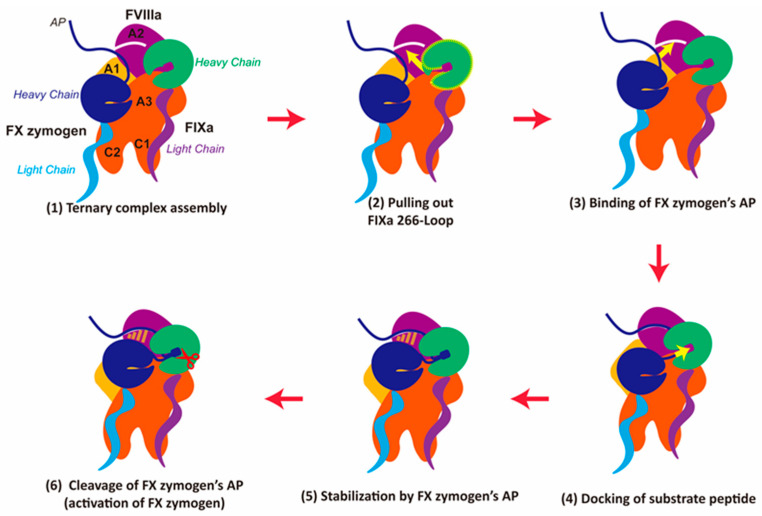
‘Binding-and-Docking’ process of FX (zymogen) cleavage by tenase. The domains of FVIIIa as well as the chains of FIXa and FX zymogen are colored differently and labeled in (1). The yellow arrows suggest the orientations of critical conformational changes. The red scissors in (6) indicate the potential cleavage of FX zymogen’s AP.

From these in silico findings, it is suggested that this hydrophobic patch on the interface between FVIIIa and FIXa functions as an exosite for FX zymogen binding. It assists in locating FX zymogen before further insertion of the cleavage site. In combination with the other discoveries above, we proposed a possible multi-step process for the functionality of the ternary complex (Figure 6): (1) ternary complex assembly, where FVIIIa, FIXa, and FX zymogen preliminarily bind together; (2) FIXa opens the 266-Loop with the help of FVIIIa’a A2 domain, which would greatly improve its activity; (3) FX zymogen binding, where the N-terminal AP assists FX zymogen to stably bind to the ternary interface by hydrophobic interactions; (4) substrate docking, where the AP of FX zymogen stabilizes substrate binding via R194’s binding to the E387 anchor at the bottom of the pocket and neighboring hydrophobic interactions; (5) accomplishment of the pre-activation state, where AP pulls the SP domain of FX zymogen closer for the stabilization of the whole ternary complex and prepares it for (6) the cleavage of FX zymogen to activate it into FXa.

Notably, our proposed pathway aligns well with that of a previously published work regarding tenase kinetics [54]. The study compared the kinetic data of FIXa activating a short peptide analog of FX zymogen’s AP and full-length FX zymogen. Through fluorescence assays, it was found that the crucial prerequisite step for the recognition and cleavage of FX zymogen’s AP is the interaction and binding of FX zymogen with FIXa. Consequently, the paper proposed a “dock-and-lock” kinetic step for the interaction between FX zymogen and the tenase binary complex. Our findings support their experimental discoveries and provide more details about the molecular mechanism behind them.

## 3. Discussion

Recently, rapid developments in computational structural modeling like AlphaFold [55,56,57] have provided more and more convenient and reliable molecular models for biological, medical, and pharmaceutical studies [58,59,60,61,62], in addition to expensive experimentally determined models. However, in the diverse world of macromolecular complexes, sometimes molecular modeling still cannot be fully accomplished by an automatic high-throughput program and needs manual vision by experts, using prior knowledge or subsequent tuning. In the case of coagulation factor complexes, the delicate interactions between the protein domains involving conformational changes caused by factor activations make this a great challenge. In this work, we fully integrated known experimental and clinical knowledge with repeated refinement and achieved the best current ternary complex of intrinsic coagulation pathway as far as we know so as to understand the structures and interactions of the coagulation factors in more details.

Our proposed ternary complex offers new insights of the molecular mechanisms involved in FVIIIa-FIXa-FX (zymogen) functionality. For the construction of the whole complex structure, there has been a proposed model for a human binary tenase complex using a prothrombinase complex [63] from Pseudonaja textilis (PDB entry: 4BXS) before the release of the template (PDB entry: 7TPP) of our AlphaFold-Multimer-based tenase binary model. In the structure with PDB entry: 4BXS, the relative position of FXa to FVa is similar to that in PDB entry: 7TPP, but the peptide that sequentially corresponds to the a2 region of FVIIIa surrounds the C-terminal helix of FXa instead of contacting FXa near the end of FVa’s A2 domain (Figure S9). However, through an experimental investigation regarding a possible contacting surface for the a2 region of the FVIIIa-FIXa tenase complex, the previous model based on template 4BXS proposed that the a2 region of FVIIIa might lie between the majority of the A2 domain and FIXa’s SP domain, which aligns well with our applied template of the human prothrombinase complex. This suggests that the a2 region in our model is properly located. In the last version of our models (Model 7), we also investigated the dynamics of the a2 region of FVIIIa, because its absence due to nonsense mutation at Y719 or E720 can cause moderate or severe coagulation deficiency [64,65]. Also, the E720K variant can cause mild coagulation deficiency according to a clinically reported case [66]. Correspondingly, we found that the segment E720-D725 (amino acid sequence: EDSYED) mediates the opening of the 266-Loop of FIXa, which can explain these cases.

In our models, the a1 region (residue 337–372) of FVIIIa was deleted mainly due to its improper folding by AlphaFold-Multimer. Nevertheless, it is also worth noticing, as there exist several clinical cases of variants in this region and the molecular mechanism is still unknown. The structure of a1 was modeled and discussed in a previous MD simulation study in an attempt to construct the structure of FVIII, in which the a1 region was described as a masking region of the 558-Loop in the zymogen that would approach the A1 domain after FVIII activation and remove its hindrance to FIXa binding. These findings are worthy insights into the structure of the a1 domain and need further support by experiments and clinical cases. In future works, we also aim to focus on this region and investigate its structural/conformational details. Although our model has already reached dynamic stability with reliable contacting surfaces in MD simulations, we also seek to include the phospholipid membrane in our system in order to provide better structural insight into the tenase complex in a physiological context.

Our proposed model also provides new structural information regarding the frequently discussed interaction between FIXa’s 333-Loop and FVIIIa’s 558-Loop. From curated FIX variant databases [50,67,68], the clinical reported cases demonstrate that every residue of 333-Loop of FIXa contains several variants that lead to severe coagulation deficiency, except for the variants at residue R338, which mostly have increased specific activity [69]. From our MD simulations, we found R338 in competition with FVIIIa’s R562 for anchoring FIXa’s E387, which assures the dynamic insertion and exclusion of FVIIIa’s R562 though the 333-Loop. Oppositely, the side chain of leucine in the R338L mutant (Padua variant) tends to be kept away by surrounding residues and not interfere with the salt bridge between FVIIIa’s R562 and FIXa’s E387, which stabilizes the “knot” conformation, keeps FVIIIa and FIXa together, and protects FVIIIa from degradation at R562. This “knotting” model seems possibly useful for explaining the activities of other R338 mutants. The reported high-activity variants Q, E, H, M, and S may also have similar molecular mechanisms, while the variant R338A’s side chain may be too short to interfere with the R562…E387 salt bridge. On the other side, in the low-activity variants, R338K has the same positive side chain that may also compete with R562 as the wild type; R338N’s short side chain is not likely to stretch out like the long side chain of the variant R338Q, but forms a hydrogen bond with its neighboring residue S339, which may rigidify part of the 338-Loop and limit the space for R562 to insert through it, similar to the R338P variant. In summary, based on our “knotting” model, although the salt bridge between the R562 of FVIIIa and E387 of FIXa through the 333-Loop is one of the key interactions for FVIIIa-FIXa binding, the interaction from residue 338 in the vicinity also plays an important role regulatory role. However, the effect of residue 338 is not simply determined by the charge or hydrophobicity of its side chain but is further affected by the surrounding residues and the flexibility of the 333-Loop, which may explain the different reported specific activities of the variants in different cell lines. As far as we know, our model is the first reported knotting mechanism for protein–protein interaction. Although we could only accomplish MD simulations on the wild type and the famous R338L variant because of limited resources, this model seems promising to explain the molecular mechanisms of a number of clinical and experimental results. We confidently expect that further computational and experimental studies on the 558-Loop of FVIIIa and 333-Loop of FIXa, especially on key residues 562, 338, 341, and 387 and other surrounding residues, may validate this hypothesis in the future.

This is also the first time utilizing in silico methods to feature the important role of FX zymogen’s AP in substrate binding to the FVIIIa-FIXa tenase. Our findings lead to a proposed multi-step binding-and-docking pathway that coincides with the investigations of previous experiments. Interestingly, we noticed a previously reported clinical case of an FX T171P mutant located in AP that causes easy bruising and bleeding [70]. According to our proposed model, this mutation may not affect the binding of FX zymogen to tenase in the pre-activation stage but may be related to the pre-maturation cleavage process of FX zymogen. We expect more computational and experimental findings on the AP of FX zymogen considering its important role.

Furthermore, our proposed model also has the potential to provide structural insights for the design of therapeutics. So far, several drugs for hemophilia A have been developed, including the clinically applied Emicizumab [71] and recently developed Mim8 [72] bispecific antibodies. The structures of both Mim8’s arms with corresponding targets have been released. We superposed the structures of FIXa and FX zymogen with their binding antibodies (PDB entry: 7AHV and 7AHU) to our proposed model, and found neither clashes nor improper distances between the two arms (Figure S10), which further supports that our proposed model contains the proper relative position of FIXa and FX zymogen and offers a reliable complex structure for future structure-based engineering of therapeutics. Our proposed complex model has great potential for future structure-based drug design targeting coagulation factors. The structural information of this ternary complex enables the design of small molecule or protein drugs that specifically bind to relevant coagulation factors in order to modulate the function of the human coagulation system. For example, researchers can screen small molecules or antibody libraries to identify drugs that structurally interfere with the formation of the intrinsic tenase complex, thereby reducing the risk of thrombosis. Additionally, researchers can utilize the conformational characteristics of coagulation factors within the complex to design drugs that enhance affinity between factors. In conclusion, the complex structure provides new opportunities for the treatment of hemophilia and thrombosis.

## 4. Materials and Methods

### 4.1. Coagulation Factor Complex Construction with Alphafold2 and Alphafold-Multimer

For constructing the FVIIIa-FIXa-FX (zymogen) ternary complex, we used AlphaFold2 [56] and AlphaFold-Multimer [55] to establish the initial complex conformations. In each run of the AlphaFold script, we have attempted to construct the monomer structures of each single coagulation factor in the FVIIIa-FIXa-FX (zymogen) complex, three binary complexes, and the whole ternary complex. We further referenced previous experimental results for screening and comparison, making minor manual structural adjustments to achieve the best modeling result. We utilized the hardware resources provided by the Shanghai Jiao Tong University supercomputing platform pi 2.0 (https://hpc.sjtu.edu.cn/ (accessed on 30 March 2025)) to run AlphaFold2 and AlphaFold-Multimer. Specifically, we used AlphaFold version 2.3.1. The alignment databases were uniref90 and uniref30 (March 2021 versions) and uniprot. The protein structure template was updated on 1 October 2022. In the final step of all modeling processes, we used GPU for structure relaxation. After obtaining the results, we ranked the structures based on the predicted Local-Distance Difference Test (pLDDT) score. We selected the top-ranked structure for subsequent comparison and analysis.

### 4.2. Molecular Dynamics Simulation of Coagulation Factor Complexes

#### 4.2.1. General

All MD simulations were performed using AMBER18 [73]. Before performing production of the MD simulations, the following phases were carried out: (1) system setup, (2) system equilibration. The AMBER FF14SB force field [74] was used for the protein. The SHAKE algorithm [75] was used to constrain the covalent bonds involving hydrogen atoms, with a 2 fs time step. The long-range electrostatic interactions were calculated using the particle mesh Ewald (PME) method [76], with a cutoff of 8 Å. The CUDA version of the PMEMD engine on either a workstation with NVIDIA RTX 3080 GPUs or a HPC node with A100 GPUs was used to accelerate the MD simulations [77]. In this work, three replicates were carried out for each production run in most of the simulations (see Table S1 for details), with the frames saved every 5000 steps for analysis. The MD simulation trajectories were visualized with PyMOL (http://www.pymol.org/pymol (accessed on 30 March 2025)) and analyzed with CPPTRAJ tools in AMBER18 [78].

#### 4.2.2. System Setup

The initial structure of each version of the complex was obtained from the last version of its MD simulation (see Table S1 for details). During MD simulations, each system was solvated in a truncated octahedron box of TIP4P-Ew water molecules [79], together with counterions (Cl^−^ and Na+) to neutralize the system.

#### 4.2.3. System Equilibration

The systems were relaxed for 6000 steps with the steepest descent minimization and 3000 steps with the conjugate gradient minimization. After minimization, the systems were heated for 20 ps and equilibrated for 20 ps in the constant-pressure and constant-temperature (NPT) ensemble at a temperature of 298.15K under standard atmospheric pressure. Lennard-Jones interactions were truncated at 8 Å. A Berendsen thermostat was used to control temperature coupling, and pressure coupling was controlled using a Berendsen barostat [80].

## 5. Summary

In this work, we tried our best to integrate known experimental and clinical knowledge into molecular models of the ternary complex of the intrinsic coagulation pathway, constructed using AlphaFold2 and AlphaFold-Multimer with repeated refinement through molecular dynamics simulations, and achieved the best ternary complex as far as we know, from which a number of novel molecular details were discovered:

1. Knot: A knot-like structure between Arg-562 of FVIIIa’s 558-Loop and the 333-Loop of FIXa contributes to FVIIIa-FIXa binding and the protection of FVIIIa from FXa cleavage, which might be the first reported case of protein–protein interactions;
2. Lid: The a2 region of FVIIIa drives the opening of FIXa’s 266-Loop (c99-Loop) and maintains its open state, which may facilitate the binding of the substrate peptide from FX zymogen;
3. Double-sided tape: The activation peptide (AP) of FX zymogen not only contributes to the binding of FX zymogen to FIXa and facilitates the activation of itself in the ternary complex but also stabilizes the ternary complex by providing double-sided interactions at the interface of the three factors.

Although our models still need validation and improvement, they are consistent with a lot of experimental results and provide good molecular mechanisms to explain the famous Padua hyperactive FIXa variant, as well as the reason why FVIIIa is a highly efficient activator of FIXa for FX zymogen activation. We expect these results to be valuable in future studies regarding the structural determination of the FVIIIa-FIXa-FX (zymogen) ternary complex, the molecular mechanisms of the intrinsic coagulation pathway, and the development of drugs/therapeutics targeting the interactions between the coagulation factors.

## Data Availability

The final AlphaFold-Multimer starting models, all MD input files, full trajectories, and analysis scripts will be deposited in Zenodo upon publication, with the accession link at https://doi.org/10.5281/zenodo.15473424. Additional data that support the findings of this study are also available from the corresponding author upon reasonable request.

## Supplementary Materials

The following supporting information can be downloaded at https://www.mdpi.com/article/10.3390/ijms26115191/s1. References [81,82] are cited in the supplementary materials.
