# Supplementary material for: The Construction of a Molecular Model for the Ternary Protein Complex of Intrinsic Coagulation Pathway Factors Provides Novel Insights for the Pathogenesis of Cross-Reactive Material Positive Coagulation Factor Mutations
Source: Int J Mol Sci. 2025 May 28;26(11):5191. doi: 10.3390/ijms26115191 (PMC12155561; doi:10.3390/ijms26115191)
Supplement: Supplementary file 1 [file ijms-26-05191-s001.zip › ijms-3591377-supplementary.pdf]

Supplementary Materials

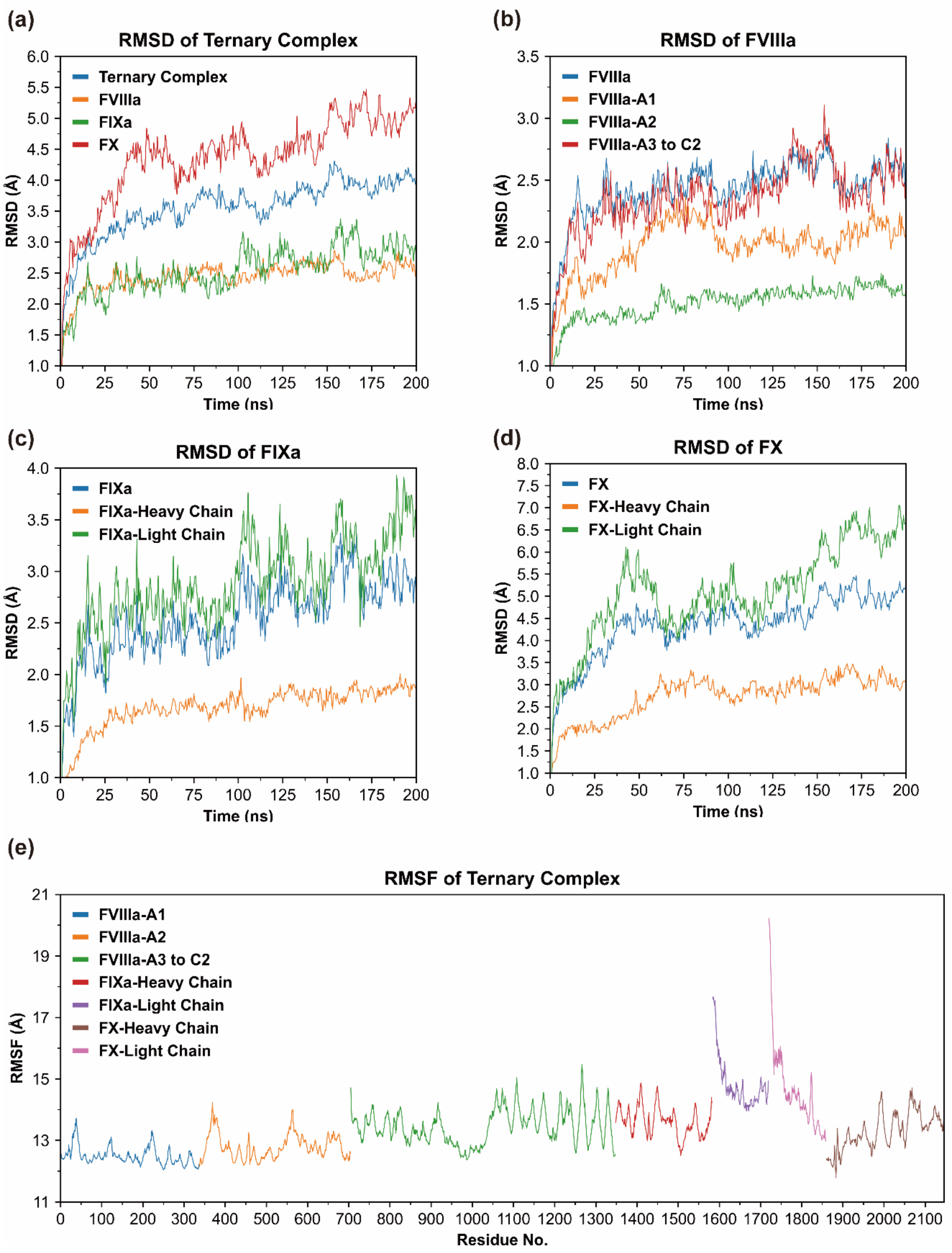

Figure S1. RMSD of the whole complex (Model 7-stable) with fluctuations in FVIIIa, FIXa, FX zymogen and RMSF of all the components in the complex.

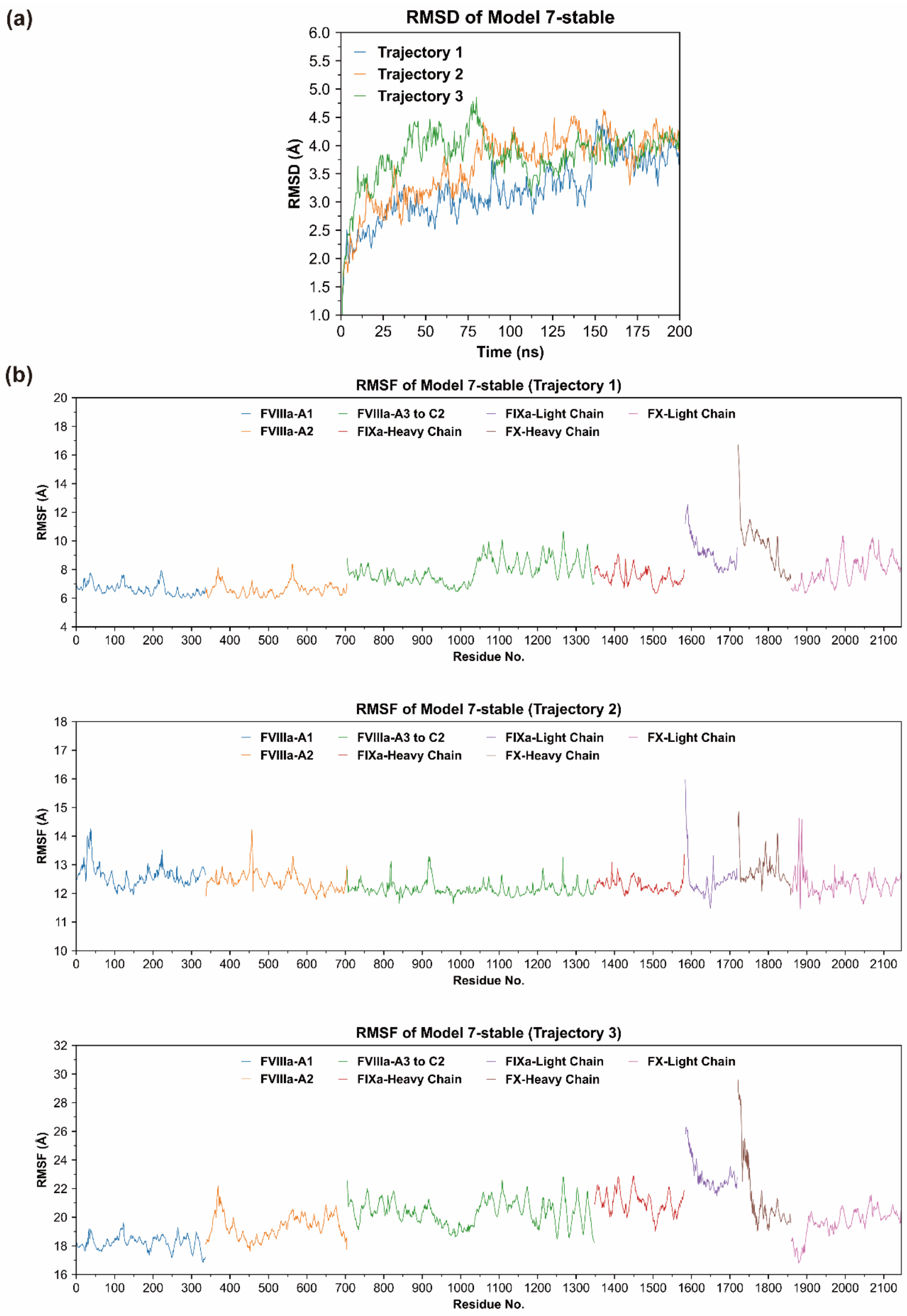

Figure S2. RMSD of the whole complex (Model 7-stable) and RMSF of all parts of the complex in three parallel trajectories.

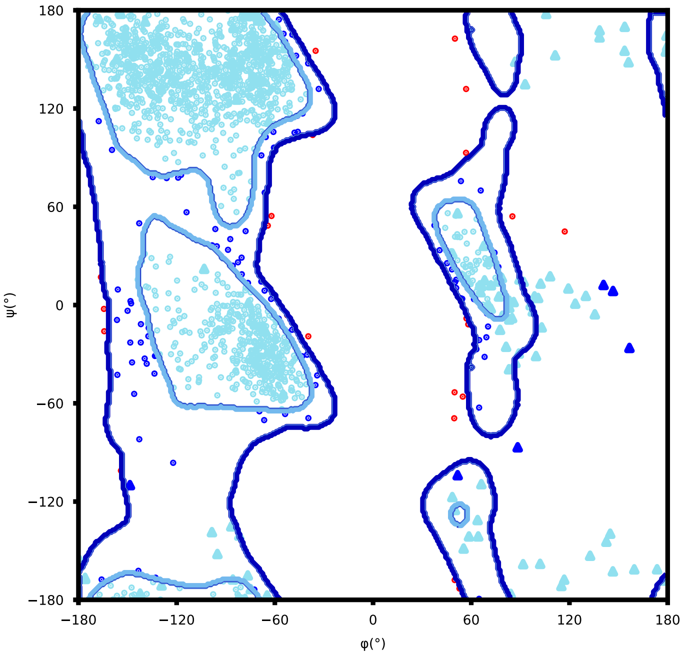

Figure S3. Ramachandran plot of Model 7-stable by RamPlot[1]. According to MolProbity Ramachandran analysis[2], 91.2% (1828/2004) of all residues were in favored (98%) regions, and 98.5% (1973/2004) of all residues were in allowed (>99.8%) regions.

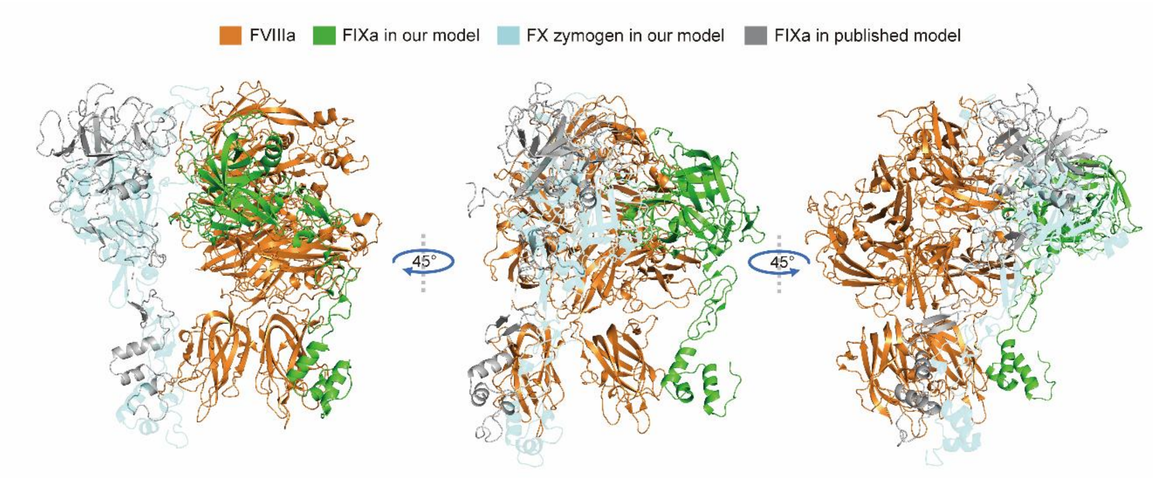

Figure S4. Comparison of our proposed model (Model 7-stable) with a previously published tenase model predicted by SAXS. For clarity, the superimposed FVIIIa structure of the SXAS model is not shown.

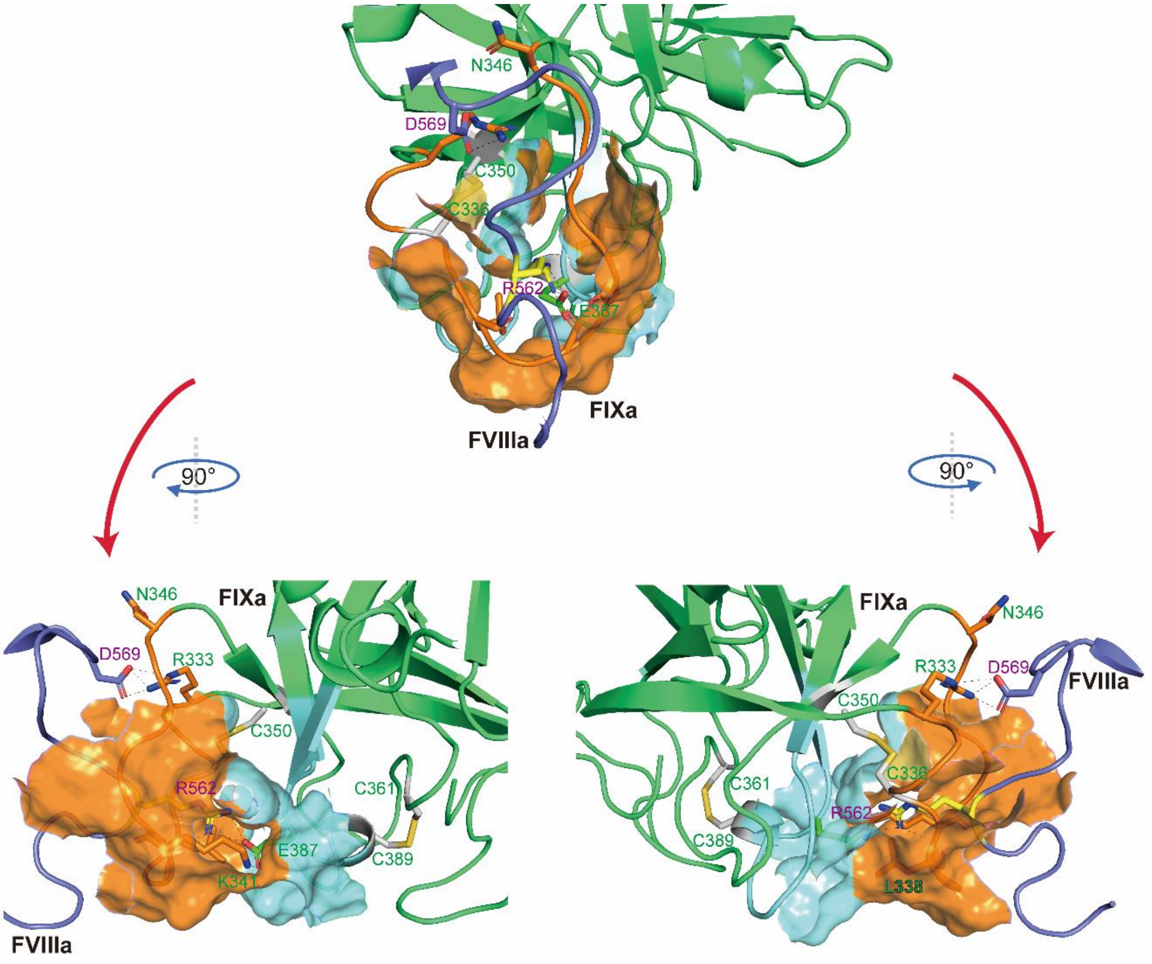

Figure S5. The space between region S384-I397 (c214-c227) and the 333-Loop of FIXa R338L variant that enables the flexibility of R562 of FVIIIa. Orange indicates FIXa's 333-Loop, while the cyan represents region S384-I397. Green represents the rest of FIXa. The transparent purple loop represents FVIIIa's 558-Loop, and the other parts of FVIIIa are not shown for clarity.

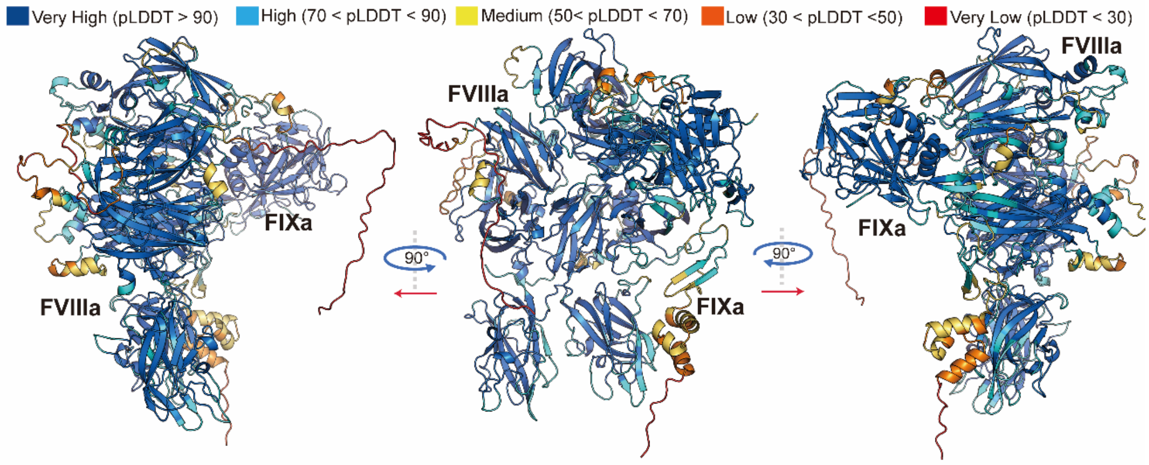

Figure S6. pLDDT of modelled FVIIIa-FIXa complex by AlphaFold2-Multimer

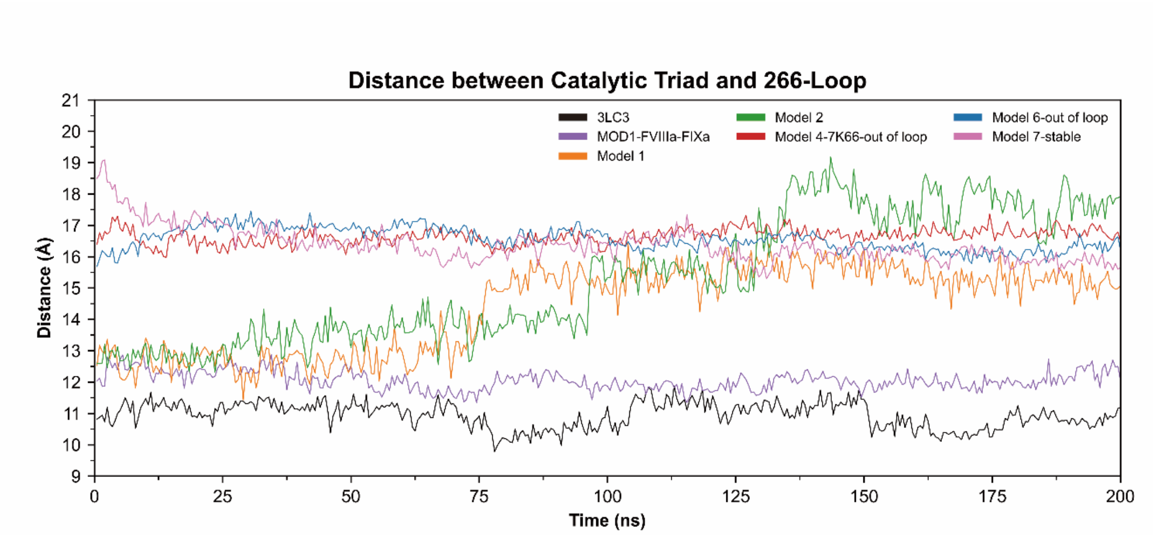

Figure S7. The distances between FIXa c99-Loop and catalytic triad in MD simulation trajectories of FIXa monomer (based on the PDB structure 3LC3) and important FVIIIa-FIXa-FX (zymogen) complex models discussed in the text

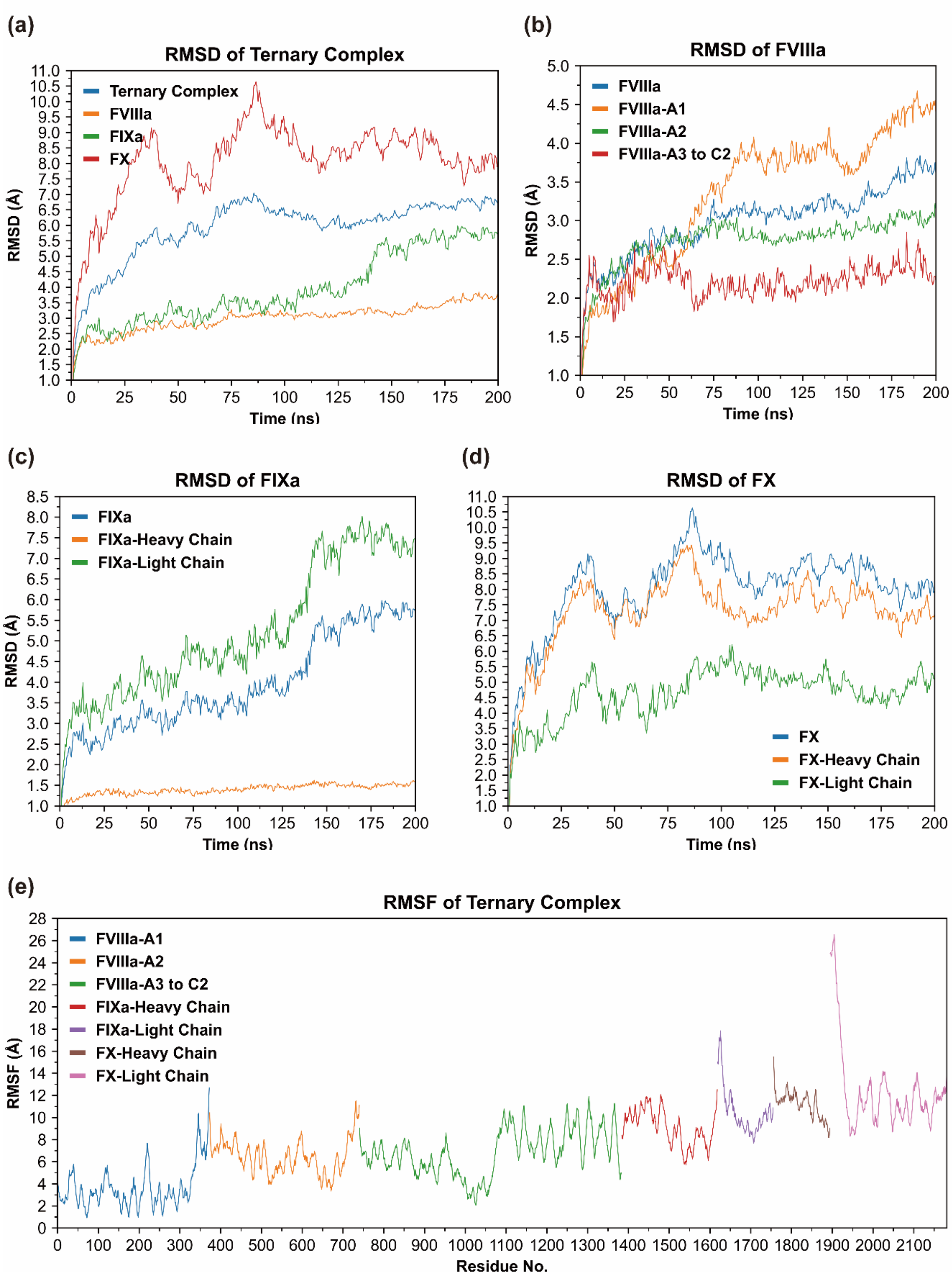

Figure S8. MD simulation results (RMSD/RMSF) of Model 6 (Model 6-out of loop).

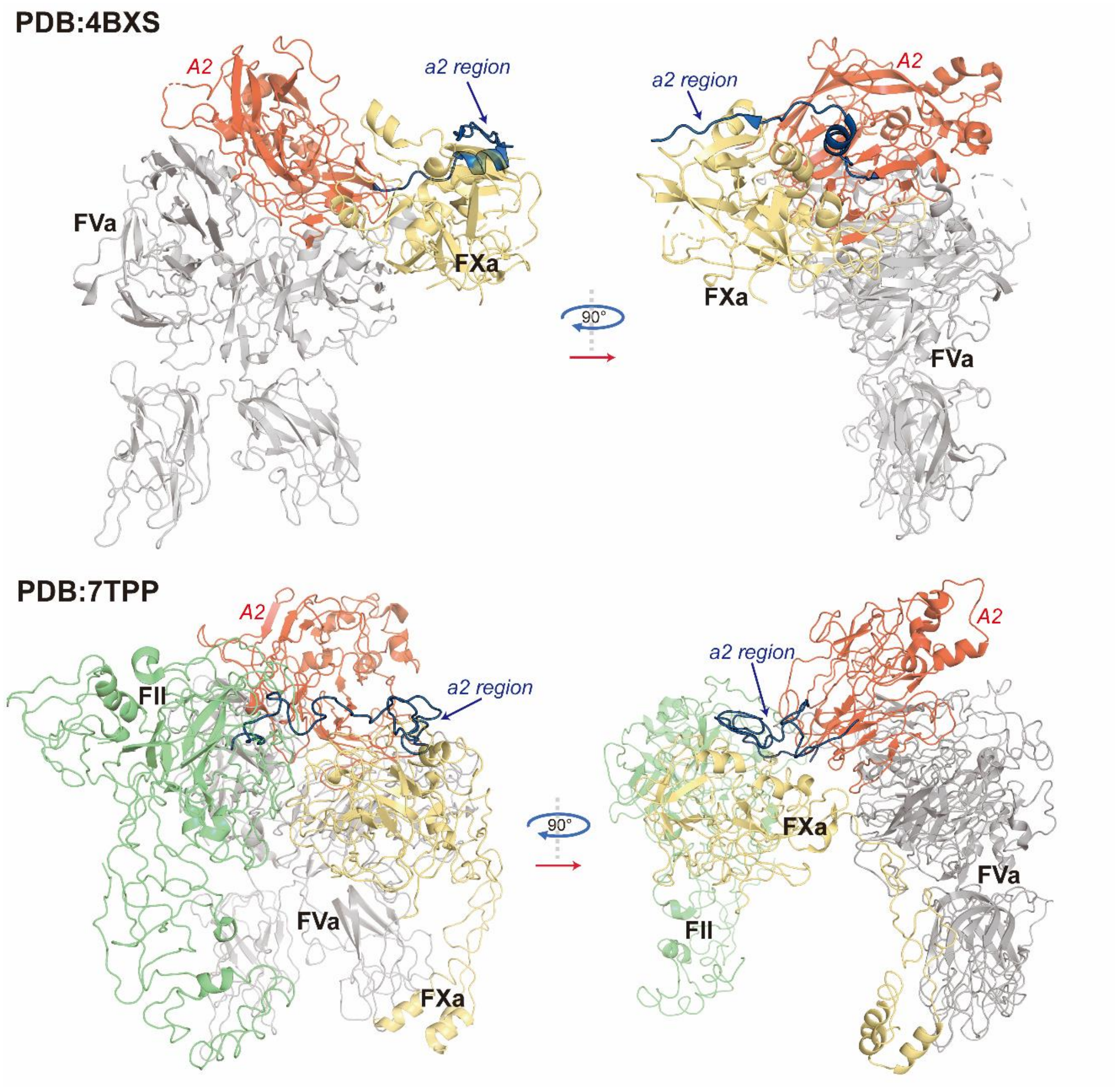

Figure S9. Comparison of two prothrombinase complex structures.

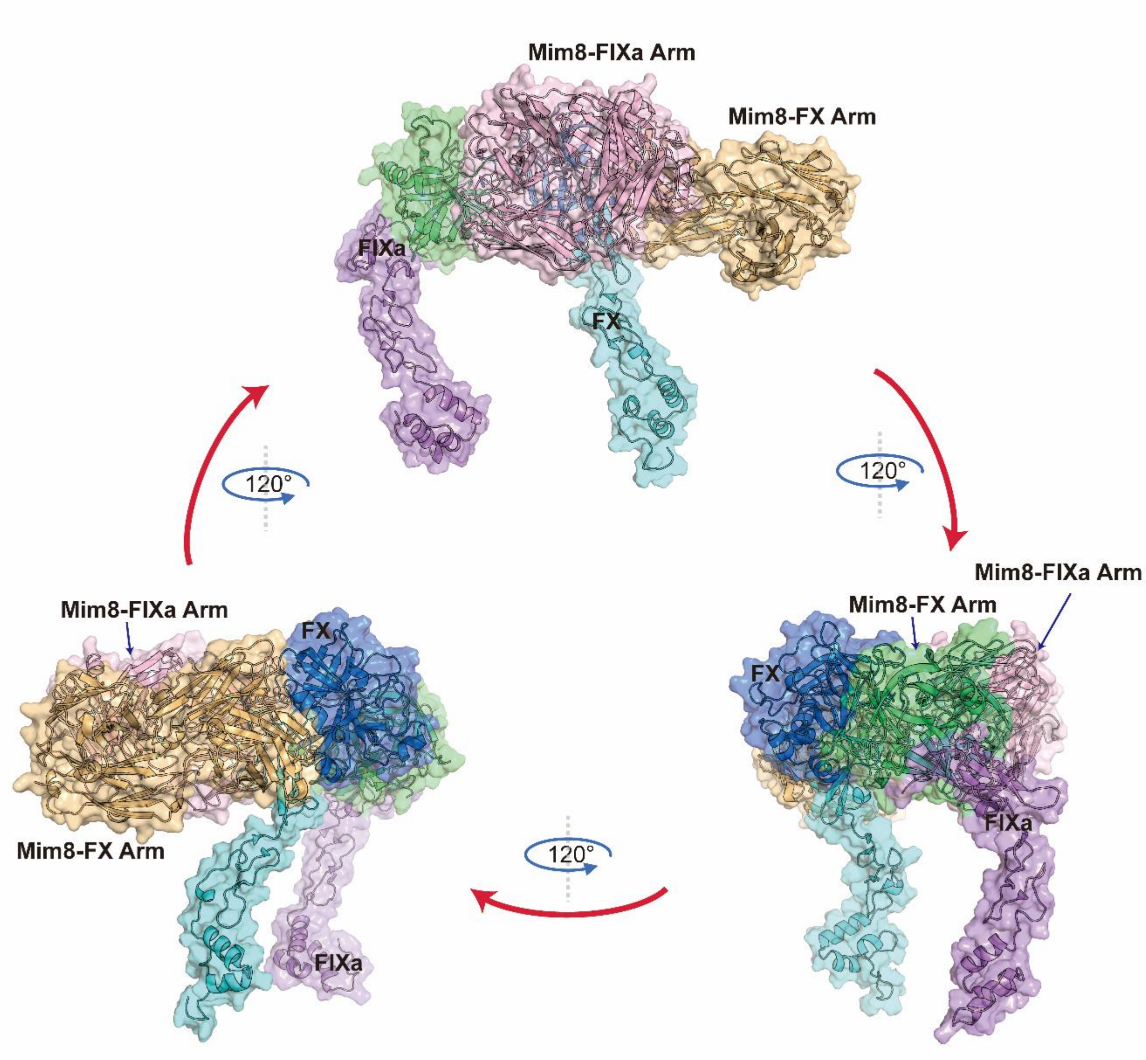

Figure S10. Proposed complex structure of Mim8 bispecific antibody's binding to FIXa and FX zymogen.

(All the supplementary figures above are in tenase-ijms-sm.docx

Table S1. Proposed Models during the Process of Ternary Complex Construction

Model Version	Model Name	No. of Trajectories	Component	MD Simulation Length (ns)	Description of Starting Conformation
0	FIXa-FVIIIa	0	FVIIIa, FIXa	0	The top rank result produced by AlphaFold-Multimer.
	**MOD1-FVIIIa-FIXa**	3	FVIIIa, FIXa	300	Structure with the C-terminal of FVIIIa's a2 region placed out of FIXa catalytic pocket.
1	Model 1	1	FVIIIa, FIXa, FX	200	Addition of FX zymogen (with 4 additional residues at C-terminal of L chain and no AP) to the complex.
2	Model 2	1	FVIIIa, FIXa, FX	200	Structure with removal of the 4 additional residues in Model 1.
3	Model 3-3AA	1	FVIIIa, FIXa, FX	100	Structure with addition of 3 more AAs to the N-terminal of AP.
	Model 3-5AA	1	FVIIIa, FIXa, FX	100	Structure with addition of 5 more AAs to the N-terminal of AP.
	Model 3-10AA	1	FVIIIa, FIXa, FX	100	Structure with addition of 10 more AAs to the N-terminal of AP.
4	Model 4-out of loop	3	FVIIIa, FIXa, FX	200	Structure with addition of full-length AP to FX zymogen and R562's conformation that is out of FIXa's 333-Loop.
	Model 4-R338L-out of loop	3	FVIIIa, FIXa, FX	100	Structure with FIXa's R338L variant, addition of full-length AP to FX zymogen and R562's conformation that is out of FIXa's 333-Loop.
	**Model 4-7K66-out of loop**	3	FVIIIa, FIXa, FX	200	Modification of FVIIIa's a1 region referring to PDB entry: 7K66 based on Model4-out of loop.
	Model 4-7K66-R338L-out of loop	3	FVIIIa, FIXa, FX	100	FIXa R338L varaint with modification of FVIIIa's a1 region referring to PDB entry: 7K66 based on Model4-out of loop.
5	**Model 5-in loop**	3	FVIIIa, FIXa, FX	100	Structure with 'in loop' conformation of FVIIIa's R562 found in the MD trajectory of Model 4-7K66-out of loop.
	Model 5-R338L-in loop	3	FVIIIa, FIXa, FX	100	FIXa R338L varaint with 'in loop' conformation of FVIIIa's R562 found in the MD trajectory of Model 4-7K66-out of loop.
6	**Model 6-out of loop**	3	FVIIIa, FIXa, FX	200	Structure with 'out of loop' conformation of FVIIIa's R562 and modification of FX zymogen position to reduce improper clash based on Moldel 5-in loop.
	Model 6-in loop	3	FVIIIa, FIXa, FX	200	Structure with 'in loop' conformation of FVIIIa's R562 and modification of FX zymogen position to reduce improper clash based on Moldel 5-in loop.
7	Model 7-out of loop	3	FVIIIa, FIXa, FX	300	Structure with removal of a1 region of FVIIIa and with the 'out of loop' conformation of FVIIIa's R562.
	Model 7-in loop	3	FVIIIa, FIXa, FX	300	Structure with removal of a1 region of FVIIIa and with the 'in loop' conformation of FVIIIa's R562.
	Model 7-R338L-in loop	3	FVIIIa, FIXa, FX	300	FIXa R338L variant with removal of a1 region of FVIIIa and with the 'in loop' conformation of FVIIIa's R562.
	**Model 7-stable**	3	FVIIIa, FIXa, FX	200	Structure with stable conformation maintained by the two-sided interaction of FX zymogen's AP discovered in Model 7-out of loop.

*Model names in bold text represent models with major modifications, and their trajectories generate the starting conformation for the next version of the models.

Table S2. Residues Contacting FX Zymogen's AP on Each Side

Residue Group 1	Residue Group 2	Frac.	Avg.	Stdev.
FX's AP-FVIIIa's A2 interactions				
Phe180(HZ)	Ile726(HG23)	0.715	3.11	0.538
Pro185(HD2)	Ile726(HD12)	0.58	3.16	0.606
Phe180(HZ)	Ser727(OG)	0.545	3.34	0.44
Thr183(HG22)	Ile726(HG22)	0.505	3.11	0.553
Thr183(CG2)	Ile726(HG22)	0.497	3.35	0.356
Thr183(HG21)	Ile726(HG22)	0.495	3.23	0.545
Thr183(HG21)	Ile726(CG2)	0.485	3.44	0.292
Pro185(HG3)	Gln565(HE21)	0.472	3.46	0.412
Thr183(HG21)	Ile726(HG23)	0.463	3.21	0.523
Gln184(O)	Ile726(HG22)	0.462	3.385	0.393
Gln184(O)	Ile726(HG21)	0.46	3.375	0.362
Thr183(HG23)	Ile726(CG2)	0.458	3.49	0.309
Gln184(O)	Ile726(CG2)	0.454	3.525	0.216
Phe180(HZ)	Ser727(HG)	0.445	3.35	0.466
Thr183(HG23)	Ile726(HG22)	0.435	3.21	0.509
Pro185(HD2)	Ile726(CD1)	0.435	3.435	0.341
Thr183(CG2)	Ile726(CG2)	0.422	3.76	0.16
Pro185(HG3)	Gln565(OE1)	0.422	3.47	0.39
Thr183(HB)	Ile726(CG2)	0.415	3.61	0.248
FX's AP-FIXa's SP interactions				
Arg194(NH1)	Cys389(HA)	1	2.707	0.208
Arg194(O)	Gly363(H)	1	1.95	0.226
Arg194(HH22)	Ser360(O)	1	2.903	0.228
Arg194(NH2)	Ser360(O)	1	2.963	0.15
Arg194(HH22)	Asp359(OD1)	1	2.233	0.265
Arg194(HH22)	Asp359(CG)	1	2.373	0.167
Arg194(O)	Gln362(HA)	1	2.66	0.249
Arg194(HH12)	Cys389(HA)	0.999	2.433	0.274
Arg194(HH22)	Asp359(OD2)	0.999	2.13	0.283
Arg194(C)	Gln362(HA)	0.998	2.983	0.213
Arg194(HH12)	Asp359(OD1)	0.998	1.917	0.228
Arg194(NH2)	Gly396(HA3)	0.997	2.803	0.248
Arg194(HD2)	Cys361(HA)	0.995	2.69	0.325
Arg194(HH21)	Ser360(OG)	0.994	2.083	0.241
Arg194(NH1)	Gly386(HA2)	0.994	2.917	0.296
Arg194(HH11)	Gly386(HA2)	0.994	2.817	0.327
Arg194(HA)	Gln362(HA)	0.984	2.707	0.294
Arg194(O)	Gly363(N)	0.984	2.86	0.148
Arg194(O)	Ser365(H)	0.983	2.553	0.401
Arg194(C)	Gly363(H)	0.977	2.857	0.205
Thr193(HA)	Trp385(HA)	0.802	2.92	0.434
Arg194(H)	Ser384(O)	0.736	2.24	0.355

Thr193(HA)	Trp385(HB2)	0.728	2.94	0.367
Leu192(O)	Trp385(HB2)	0.728	2.753	0.31
Thr193(HA)	Trp385(HB3)	0.727	2.513	0.33
Thr193(HA)	Ser384(O)	0.71	2.923	0.298
Glu186(O)	Phe342(H)	0.598	2.693	0.491
Leu192(HB3)	Trp385(HE3)	0.551	3	0.43
Glu186(HB3)	Lys341(HG3)	0.488	2.893	0.499
FX's AP-FX's SP interactions				
Gln182(O)	Gln255(HE21)	0.752	2.053	0.371
Gln182(O)	Gln255(NE2)	0.736	2.923	0.21
Gln182(H)	Gln255(HE21)	0.708	2.97	0.287
Glu186(OE2)	Arg336(HH11)	0.706	2.44	0.643
Glu186(OE1)	Arg336(HH11)	0.693	2.51	0.625
Glu186(OE1)	Arg336(HH22)	0.689	2.34	0.571
Glu186(OE2)	Arg336(HH22)	0.676	2.503	0.579
Gln182(H)	Gln255(OE1)	0.664	2.73	0.378
Asn181(H)	Gln255(OE1)	0.642	2.283	0.191
Phe180(HB2)	Gln255(HE21)	0.636	2.993	0.421
Asn181(HD22)	Glu254(O)	0.629	2.44	0.634
Gln184(HE22)	Glu257(OE2)	0.545	2.913	0.555
Gln184(HE22)	Glu257(OE1)	0.54	2.573	0.618
Gln184(HE21)	Arg251(HH21)	0.516	2.913	0.365
Gln182(OE1)	Asn252(HD21)	0.509	2.103	0.443
Gln184(HE22)	Arg251(HH22)	0.481	2.747	0.401
Gln182(OE1)	Asn252(ND2)	0.476	2.947	0.21

References

1. Kumar, M. and R. Rathore, *RamPlot : a webserver to draw 2D, 3D and assorted Ramachandran (φ, ψ) maps.* Journal of Applied Crystallography, 2025. **58**.
2. Williams, C.J., et al., *MolProbity: More and better reference data for improved all-atom structure validation.* Protein Sci, 2018. **27**(1): p. 293-315.
